# Navigating the Groove: A Unique Case of Groove Pancreatitis

**DOI:** 10.7759/cureus.60471

**Published:** 2024-05-16

**Authors:** Oshin Rai, Anvit D Reddy, Vanshika Tripathi, Natalie Shaykh, Noor Marji, Ghania Masri, Bruno Ribeiro

**Affiliations:** 1 Internal Medicine, University of Florida College of Medicine-Jacksonville, Jacksonville, USA; 2 Pathology and Laboratory Medicine, University of Florida College of Medicine-Jacksonville, Jacksonville, USA; 3 Gastroenterology, University of Florida College of Medicine-Jacksonville, Jacksonville, USA

**Keywords:** hemangioma, paraduodenal pancreatitis, whipple’s pancreaticoduodenectomy, chronic pancreatitis, groove pancreatitis

## Abstract

Groove pancreatitis (GP) is an uncommon form of chronic pancreatitis (CP) that affects the area between the duodenum, the head of the pancreas, and the common bile duct (CBD), which is known as the pancreaticoduodenal groove. Our case is based on a 68-year-old male with a past medical history of alcohol use disorder and a 50-pack-year smoking history who presented with nausea, vomiting, and poor oral intake. Computed tomography (CT) of the abdomen and pelvis showed gastric outlet obstruction due to a 6.0 cm mass in the pancreatic groove and the second portion of the duodenum, with dilation of the pancreatic, intrahepatic, and extrahepatic biliary ducts. In order to rule out malignancy and evaluate the acute symptoms, the patient underwent an open pancreaticoduodenectomy (PD). Pathologic findings and negative tumor markers confirmed GP. This case highlights a rare form of CP that symptomatically and radiographically mimics malignancy, but is benign.

## Introduction

Groove pancreatitis (GP) is related to chronic pancreatitis (CP) affecting an area known as “the groove” situated between the pancreatic head, duodenum, and common bile duct (CBD) [1]. Typically diagnosed in individuals between their fifth and sixth decades of life, it shows a higher incidence in males [2]. It is still an uncommon diagnosis, and therefore, the incidence is not well-documented [3,4]. Although the exact etiology of GP remains elusive, it is associated with excessive alcohol intake, smoking, peptic ulcer disease, biliary issues, gastric resection, heterotopic pancreas, and blockages of the minor papilla, whether anatomical or functional [5]. Most patients with GP describe intense upper abdominal pain, nausea, and recurrent postprandial vomiting that can last from weeks to several years, resulting in weight loss [6,7]. A characteristic feature of GP is duodenal stenosis which, when severe, leads to obstructive symptoms [6]. While tubular stenosis of the CBD is a common occurrence, obstructive jaundice is seldom observed and typically presents later in the clinical course compared to cases of pancreatic adenocarcinoma (PA) [6]. 

The radiological features of GP can differ depending on whether it is the segmental form or the pure form of the process. The segmental form can involve both the pancreaticoduodenal groove and portions of the pancreas, whereas in the pure subset, only the groove is affected. Computed tomography (CT) and magnetic resonance imaging (MRI) can reveal inflammation, fat-stranding, cystic changes, and soft-tissue fibrosis in pure GP. In segmental GP, the pancreas head can be enlarged and can obscure evaluation of the pancreaticoduodenal groove on imaging. The segmental form of GP is often difficult to discern from a pancreatic head mass or malignant process based on radiographic findings. In either pure or segmental GP, nearby structures can help characterize the disease process further. For example, in both forms, the CBD is usually narrowed, typically in a smooth and tapered fashion. If the changes are acute, the pancreatic duct also follows a smooth and gradual tapering pattern. Comparatively, in the chronic setting, the pancreatic duct will have progressive narrowing, ductal beading, or irregularity paired with parenchymal changes such as calcifications and fibrosis [1].

Management of GP varies depending on if it can be distinguished from malignancy. In the event that it can be correctly identified, supportive therapies can be chosen. However, due to clinical and radiographic ambiguity, patients commonly undergo pancreaticoduodenectomy (PD) [1]. 

## Case presentation

**Figure 1:**
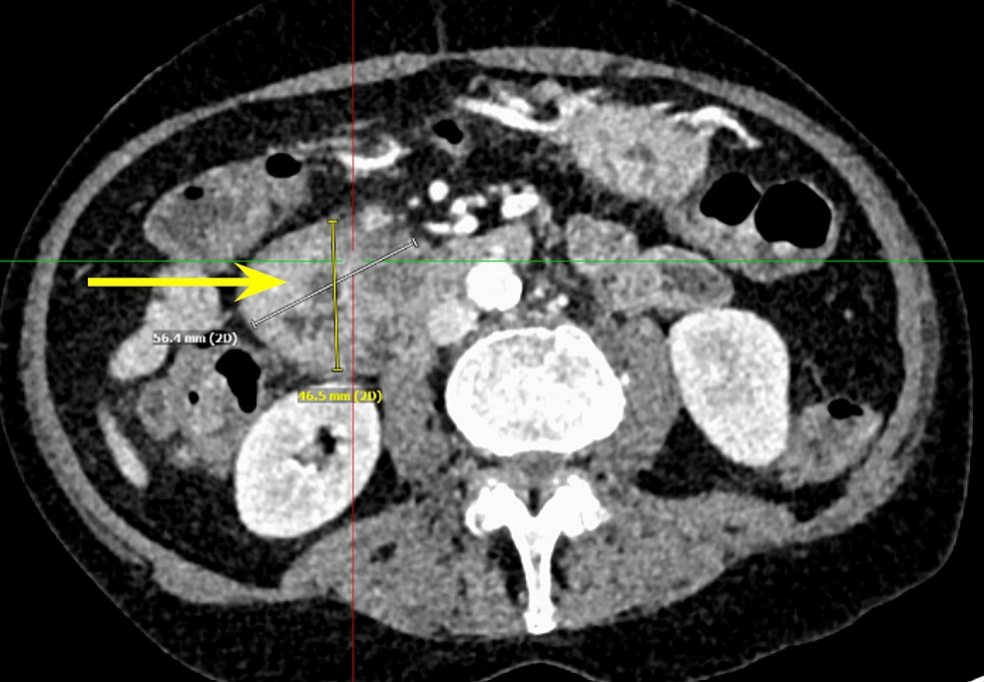
Computed tomography of the abdomen and pelvis showing a pancreatic mass measuring grossly 5.6 cm x 4.6 cm in the anterior-posterior view or transverse plane.

**Figure 2:**
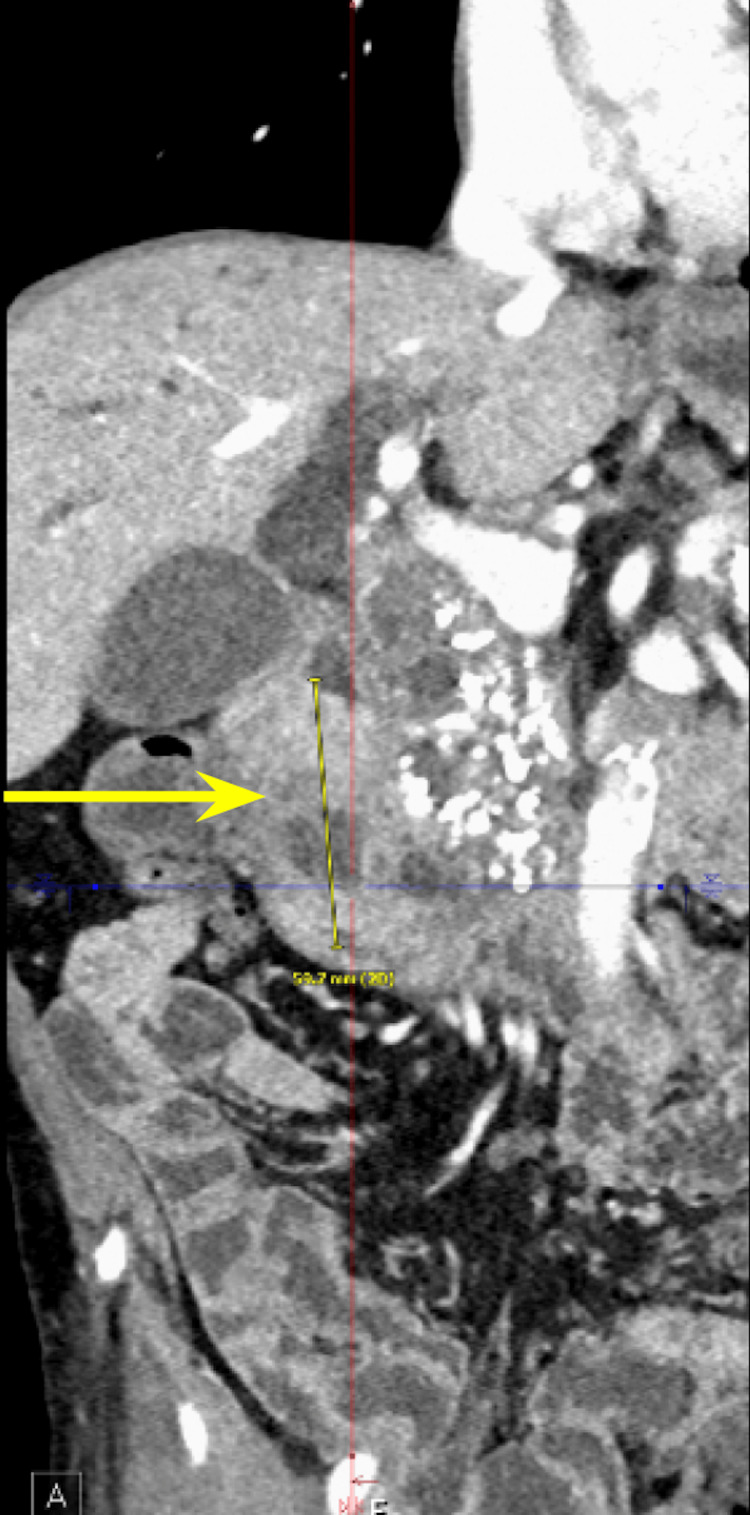
Computed tomography of the abdomen and pelvis showing a pancreatic mass measuring grossly 5.6 cm in the coronal plane.

We present a case of a 68-year-old male with a past medical history of prostate cancer requiring radical prostatectomy, hypertension, hyperlipidemia, alcohol use disorder, and 50-pack-year smoking history who presented with several weeks of anorexia, dyspnea on exertion, generalized weakness, nausea, and vomiting. He also noted an unintentional 20-pound weight loss over the last several months. Vital signs were within normal limits with blood pressure of 118/80 mmHg, pulse of 98 beats per minute, respiratory rate of 16 while saturating 100% on room air, and temperature of 98.8°F. Physical exam was remarkable for a cachetic, ill-appearing male in no acute distress. Laboratory evaluation was significant for acute kidney injury (creatinine (Cr) of 2.89 mg/dL from baseline of 0.5-0.6 mg/dL; reference range: 0.51-0.96 mg/d), mild transaminitis with aspartate transferase (AST) of 46 IU/L (reference range: 14-33 IU/L), anion gap of 28 mmol/L (reference range: 4-16 mmol/L), lactic acidosis of 8 mmol/L (reference range: <2 mmol/L), and beta-hydroxybutyrate of 4 mmol/L (reference range: 0.5 mmol/L). CT of the abdomen and pelvis showed three main findings. The first was a large 6.0 cm heterogeneous mass located in the second portion of the duodenum and pancreatic groove (Figures 1-2).

**Figure 3:**
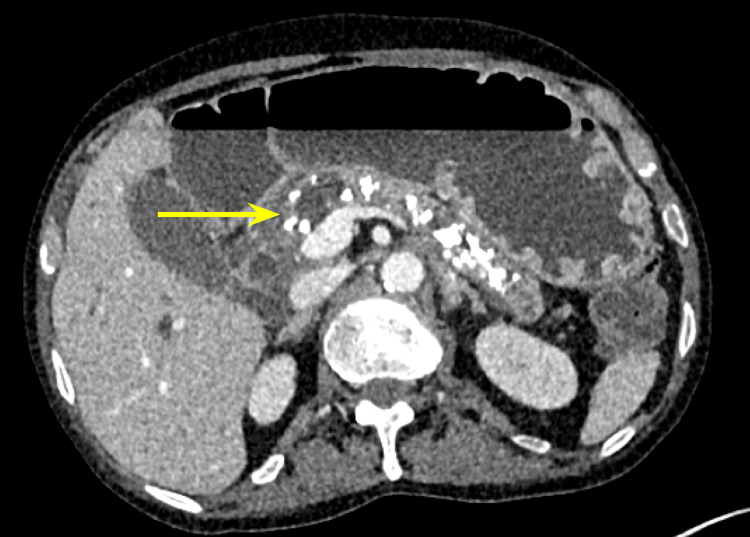
Chronic pancreatitis evidenced by heavy calcification on computed tomography of the abdomen and pelvis.

**Figure 4:**
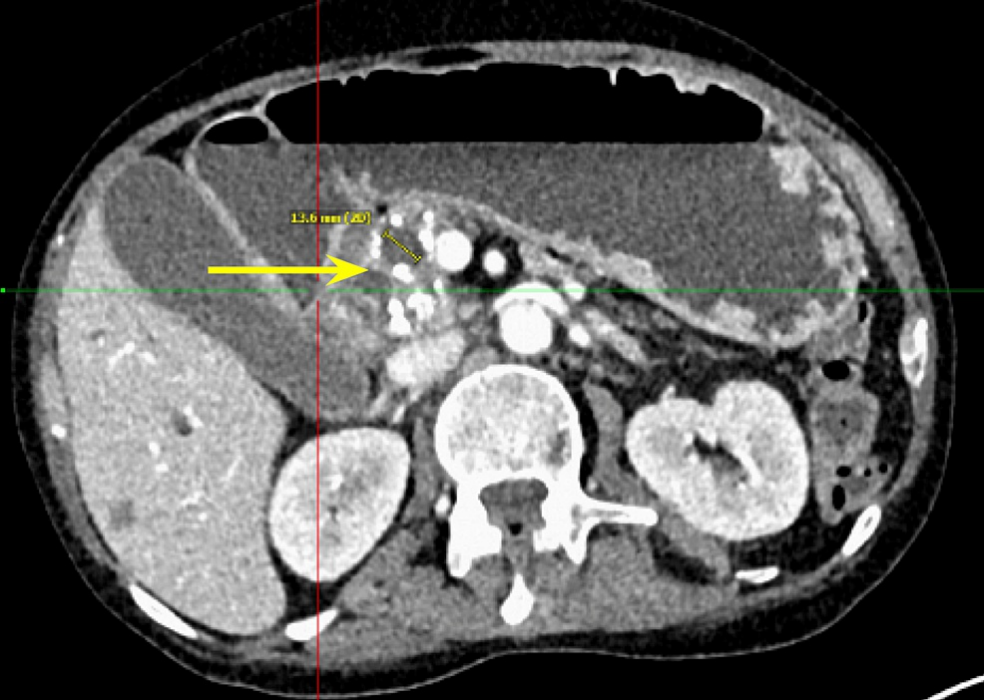
Computed tomography of the abdomen and pelvis showing dilation of the pancreatic duct, approximately 1.3 cm.

Additionally, there were multiple coarse calcifications throughout the pancreas, consistent with CP (Figure 3). Lastly, the mass was causing significant dilation of the pancreatic (Figure 4), intrahepatic, and extrahepatic biliary ducts. 

**Figure 5:**
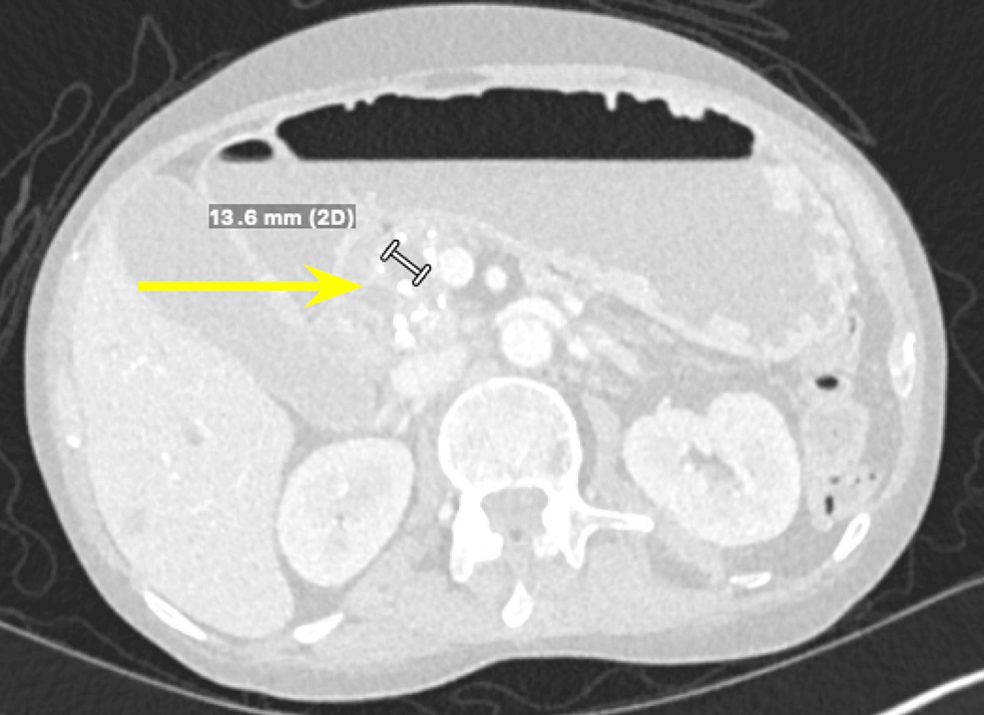
Computed tomography of the abdomen and pelvis showing a hepatic lesion, measuring 1.36 cm.

Tumor markers including cancer antigen 19-9 (CA 19-9), carcinoembryonic antigen (CEA), and alpha-fetoprotein were all within normal limits. Multiple lymph nodes were enlarged, with the peripancreatic and mesenteric lymph nodes being greater than 8 mm and the portacaval lymph node measuring 9 mm. Furthermore, there was a lesion noted on the liver (Figure 5), later classified through liver MRI as a benign hemangioma.

 

**Figure 6:**
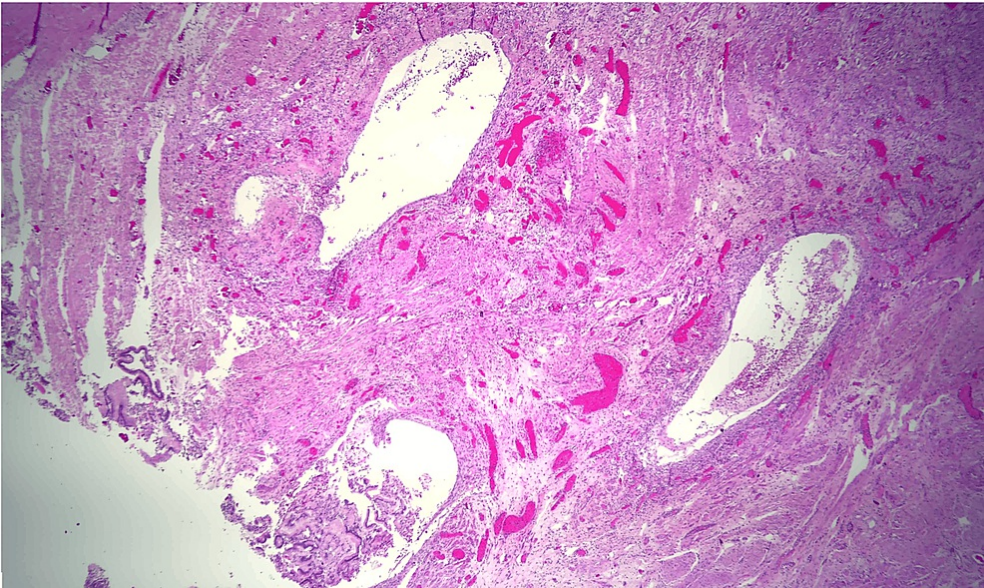
Groove pancreatitis (paraduodenal pancreatitis) showing pseudocysts (lined by granulation tissue) of the duodenal wall, fibrosis, and chronic inflammation.

**Figure 7:**
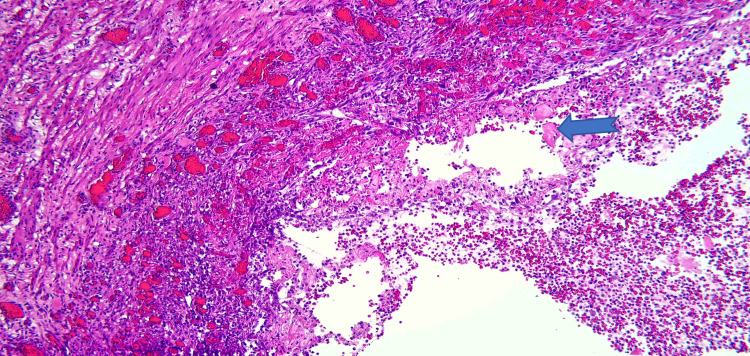
High-power view of the pseudocyst which showed granulation tissue and stone fragments (blue arrow).

**Figure 8:**
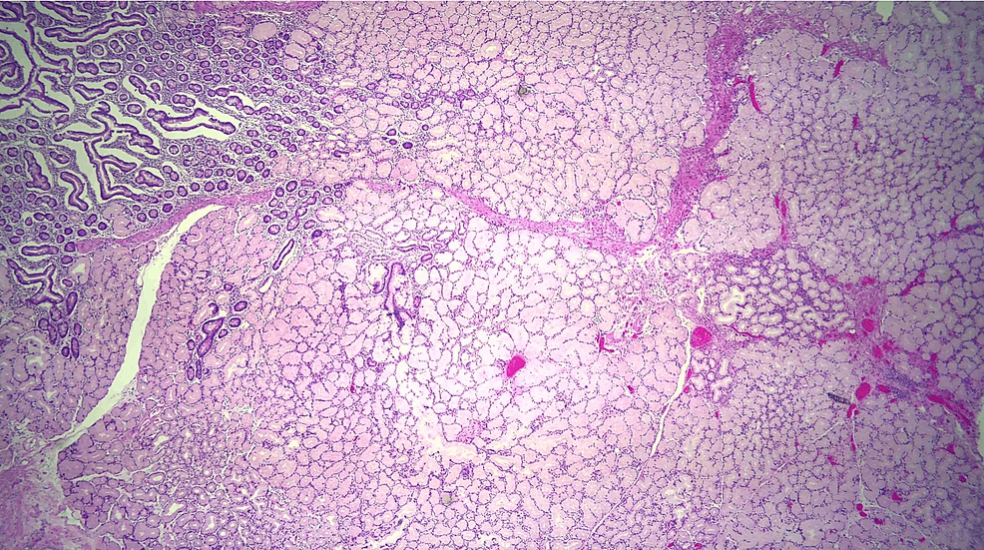
Brunner gland hyperplasia.

Due to the patient’s symptoms, risk factors, and degree of obstruction, these findings were concerning for possible metastasis. Both gastroenterology and general surgery were consulted for consideration of either endoscopic ultrasound with fine-needle aspiration or open surgical biopsy of the mass. Ultimately after multidisciplinary discussion, general surgery was planned for a biopsy of the mass and management with PD. Pathology revealed Brunner gland hyperplasia, pseudocysts with granulation tissue, chronic inflammation, and proteinaceous material consistent with GP (Figures 6-8). MRI of the liver deemed the lesion to be a benign hemangioma. Additionally, cluster of differentiation 31 (CD31), human herpesvirus-8 (HHV-8), and *Helicobacter pylori* immunostaining were all negative. He did well postoperatively and was discharged with bilateral Jackson-Pratt drains. At his outpatient follow-up, he endorsed improvement in appetite and resolution of his symptoms. 

## Discussion

Acute pancreatitis (AP) is a common hospital diagnosis in the United States, with an estimated incidence of 600-700 cases per 100,000 persons per year [8]. Common etiologies include alcohol use, gallstones, and hypertriglyceridemia [8]. CP is diagnosed in patients with recurrent bouts of AP, which presents with nausea, vomiting, poor oral intake, and radiographic findings suggestive of CP. It is less common than AP with an estimated incidence of eight per 100,000 persons [9]. GP is a rare cause of CP, with no clear incidence or prevalence reported due to limited data. The condition is often mistaken for PA and has an incidence of roughly two per 100,000 people in the United States, making it more common than GP [10]. Distinguishing between GP and PA poses challenges in the clinical setting, as they display similar clinical features, radiologic findings, and gross pathological characteristics including prominent scarring and indistinct borders [6]. Missing a diagnosis of PA can be potentially devastating, as the estimated five-year mortality of PA is 13% [10]. All of these factors are why patients with GP oftentimes end up undergoing an open PD, even though the process itself is managed with supportive treatment.

Laboratory evaluation with CA 19-9 helps differentiate between GP and PA. Elevation of CA 19-9 levels has a sensitivity and specificity of 79%-81% and 82%-90%, respectively, for the diagnosis of PA. However, limitations of this marker include low positive predictive value (0.5%-0.9%), false positivity in obstructive jaundice (10%-60%), and false negatives in Lewis negative phenotypes (5%-10%) [11]. A study from Tarvainen et al. noted that one-third of patients with GP had elevations in CA 19-9 and CEA [12]. Some studies have reported significantly higher CA 19-9 values in PA compared to GP, but there is not enough data to delineate between the two [12]. This patient had CA 19-9 and CEA levels within normal limits, which favored a nonmalignant diagnosis.

Given the patient’s initial presentation and workup, malignancy was high on the differential. This was due to his smoking history, 20-pound unintentional weight loss and an incidental finding of a suspicious lesion on the liver. This liver lesion was first noted on the CT abdomen and pelvis, but further characterization on MRI deemed it to be a benign hemangioma. According to Raman et al., patients who have jaundice due to distal CBD narrowing coupled with chronic weight loss favor a diagnosis of underlying malignancy [1]. Our patient, in contrast, had imaging which showed irregular CBD dilation.

Management of GP is variable and depends on the certainty that malignancy is not present. If GP is suspected, patients are managed symptomatically with goals of promoting weight gain. In cases of gastric outlet obstruction from mass effect, bypass procedures such as gastrojejunostomy with the addition of choledochojejunostomy for biliary involvement can be performed. The disadvantage of these therapies is that symptoms can persist, progress, and worsen, prompting further invasive intervention. Additionally, with no definitive histology, malignancy cannot completely be excluded. The definitive management of GP is still a PD, as the mass can be fully resected with a pathological diagnosis. Although PD is an extensive surgical procedure with morbidity and mortality rates of 5% and 2%, respectively [13], it allows for curative treatment of GP. With malignancy on the differential, our patient also underwent 19 lymph node biopsies, which were all negative for metastatic tumor cells.

## Conclusions

GP is a form of CP that is not commonly seen and can be a challenging diagnosis with a broad differential. Symptoms can be based on chronicity but typically include abdominal pain, nausea, weight loss, and if more acute even postprandial emesis. Diagnosis is made through imaging such as CT or MRI, but ultimately management is guided clinically. Management can include therapy targeted to relieve symptoms or bypass procedures in the setting of gastric outlet obstruction. Oftentimes, due to the suspicion of malignancy, patients are definitively treated with PD. Our patient had many risk factors for malignancy and underwent an open PD with biopsy but was ultimately found to have a benign process.
